# Physical activity and gender buffer the association of retirement with functional impairment in Ghana

**DOI:** 10.1038/s41598-022-17178-z

**Published:** 2022-07-27

**Authors:** Razak M. Gyasi, Padmore Adusei Amoah, Seth Agyemang, Lawrencia Pokua Siaw, Foster Frempong, Ritu Rani, David R. Phillips

**Affiliations:** 1 Aging and Development Unit, African Population and Health Research Center, Manga Close, Off-Kirawa Road, P. O. Box 10787-00100, Nairobi, Kenya; 2 National Centre for Naturopathic Medicine (NCNM), Faculty of Health, Southern Cross University, Lismore, NSW Australia; 3 School of Graduate Studies, Department of Applied Psychology, Asia Pacific Institute of Aging Studies, Lingnan University, Tuen Mun, Hong Kong; 4 Department of Geography and Rural Development, Kwame Nkrumah University of Science and Technology, Kumasi, Ghana; 5 International Institute for Population Sciences, Mumbai, India; 6 Department of Sociology and Social Policy, Lingnan University, Tuen Mun, Hong Kong

**Keywords:** Diseases, Health occupations, Rheumatology

## Abstract

Females on average live longer but with higher rates of functional impairment and lower physical and economic activities than men. However, research linking retirement to functional impairment and the modifying role of gender and physical activity (PA) is limited especially in low- and middle-income countries. This paper examines the association between retirement and functional impairment in Ghana and evaluates the effect modification of the association by gender and PA. The sample included 1201 adults aged ≥ 50 years from a population-based study. Functional impairment was assessed with the activities of daily living scale. Ordinary least squares regression models adjusted for confounding variables and estimated gender-wise and PA heterogeneity effect of retirement on functional impairment. Regressions showed that retirement predicted an increase in functional impairment score in the full sample (*β* = .76, *p* < .001) and in men (*β* = 1.96, *p* < .001), but not in women. Interestingly, retirement significantly increased functional impairment in ≥ 65 age cohort (full sample: *β* = .71, *p* < .005; men: *β* = 1.86, *p* < .001) although not in women. However, the effect was significantly moderated by PA such that retirement × PA predicted a decrease in functional impairment in the full sample (*β* = −.81, *p* < .005) and the ≥ 65 age group (*β* = −.43, *p* < .005). Functional impairment risk of retirement is gender-specific, but PA buffers the relationship. Retirement is generally commonplace, but these findings imply that promoting PA may hold promise for addressing functional impairment in old age. Attending to the physical health needs of men during retirement should be a social policy priority.

## Introduction

Functional impairment, defined as the inability of persons to carry out certain functions in their daily lives due to illnesses and diseases^1^ and the tendency for reduced physical activities on retirement, in combination pose significant health and contemporary social policy challenge. In sub-Saharan Africa (SSA), an estimated 43% of persons 60 years or over report disabilities^2,3^, and difficulty undertaking ADL, mainly due to contextual risk factors^4,5^. Functional impairment is a leading cause of disability, long-term severe care needs, and medical costs^6^.  However, the right for older people to live and participate socially is emphasized by the United Nations Convention on the Right of Persons with Disabilities^7^ and for successful aging^8^. In this context, the concern about apparently rising functional impairment in rapidly aging adults in SSA stimulates a renewed interest in identifying the risk factors associated with functional impairment.

Research in Western and Asian societies has suggested effects of retirement on subsequent health outcomes, including cognitive decline^9,10^, depression^11^, psychological state^12^, and functional impairment. Findings are not clear cut and a systematic review found inconsistencies in the association of retirement with functional impairment^13^. Dave et al.^14^ relate retirement to a 5–16% increase in difficulty in ADL. Some studies suggest that the transition into retirement alleviates physical limitations and contributes to better health^15,16^ while others found no associations between the variables^17,18^. Retirement may be linked to functional impairment via several mechanisms. For example, retirement may invoke changes in lifestyle behavior, including physical activity (PA) and social participation^19^ which can potentially stimulate body-mind interactions for healthy living. Further, the anticipation of disengagement and uncertainty in retirement-induced economic hardships can lead to stress responses, contributing to depression and anxiety^20^ and subsequently amplifying functional limitations^21^.

Several authors have identified heterogeneity in the aging population in terms of physical health outcomes and survival and have noted marked gender differences^22,23^. The prevalence of functional impairment, including ADL and mobility limitations, is generally higher in women, partly due to women’s greater socioeconomic disadvantage (especially in low- and middle-income countries), their chances of surviving longer, and other biological disparities^6,24^. With aging populations generally including more females than males and with often established gender differences in the nature of work and retirement, it is essential to understand whether any functional impairment effect of retirement varies between gender subgroups. Studies have indicated that females have an increased risk of retiring earlier than males^25^. Others such as Radl^26^, on the other hand, note that in countries with low female labor participation rates, females retire later than males. Yet other studies suggest no apparent differences in retirement between the gender subgroups as males and females have markedly different socioeconomic positions and retirement patterns^27–29^. It has also been shown that although physical activity (PA) is a salient psychosocial resource for health through coping with stressful life events^30^, PA declines after retirement^9^. As a social determinant of health, PA may relate to life course transitions and improve functional well-being^31^. Cross-sectional and prospective studies report various levels of PA associated with better functional abilities^32–35^. For example, longitudinal data from the Frailty, Dynapenia, and Sarcopenia study among Mexican adults found that moderate to vigorous PA intensity significantly reduced difficulties in ADL and physical performance^36^. However, we are unaware of any study exploring the effect modification of the association between retirement and functional impairment by PA, particularly in low- and middle-income countries. In SSA in particular, whether the risk of functional impairment after retirement differs across gender subgroups remains unclear.

This study employs exploratory survey data to examine the gender-specific associations between retirement and functional impairment in Ghana and evaluates the potential modifying effect of PA in these associations. Despite the rising and gendered patterns of life expectancy, the average retirement age is declining locally due to increasing trends in voluntary retirement^37^. For example, the Ghana Statistical Service^38^ has shown a reduction in the average retirement age from 60 years to about 58 years for males and 55 years for females. This is related to the factors that motivate premature retirement, including the perception of worsening health status, rising burden of disease, increasing caregiving burden, and other socioeconomic events^11,39^. Our study is important to improve understanding of gender-based retirement and functional status with relevant implications for public health policy. We hypothesized: (1) that the risks of functional impairment increase after retirement; (2) that the impact of retirement on functional impairment would differ by gender; (3) that PA would modify the association between retirement and functional impairment.

## Results

**Figure 1:**
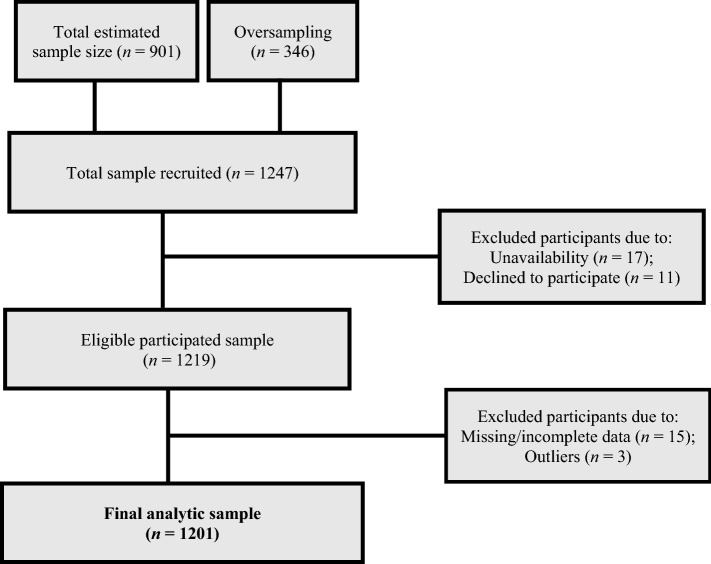
Flow chart of the selection of study participants.

After exclusion criteria were applied, 1201 respondents were included in the final analytical cohort (Fig. 1). About 63% were women, and the mean age was 66.2 years (SD = 11.9). Most participants were not married (65%), lived in urban areas (55%), and had up to primary education (86%). Income levels were low with an average of GH¢308 (SD = 338.89) (US$64). Almost 50% of the sample perceived their overall health as poor/fair, while the mean psychological distress, functional impairment, and physical activity scores were 15.91 (SD = 4.66), 13.70 (SD = 5.09), and 6.10 (SD = 2.68) respectively. About 16% of the sample were retired and the mean social connectedness score was 6.09 (SD = 2.6. The characteristics of the sample are summarized in Table 1.

**Table 1:** Characteristics of the sample.

Variable	*N* = 1201	(%)	Mean	(± SD)	Range
Retired	188	(15.7)			
PA score			6.10	(± 2.68)	0–21
Mean age (in years)			66.15	(± 11.85)	50–111
Women	759	(63.3)			
Rural residence	540	(45.0)			
Marital status: Married	521	(44.3)			
Marital status: Widow	473	(40.3)			
Marital status: Divorced/separated	181	(15.4)			
Primary/no education	1034	(86.2)			
Secondary education	104	(8.7)			
Higher education	62	(5.2)			
Income level (in Ghana Cedis)			308.18	(± 338.89)	100–4000
Social connectedness			6.09	(± 2.679)	0–20
SRH: Excellent/Very Good	239	(19.9)			
SRH: Good	369	(30.8)			
SRH: Fair	348	(29.0)			
SRH: Poor	244	(20.3)			
Psychological distress score			15.91	(± 4.66)	0–40
FI score			13.70	(± 5.09)	0–18

FI—functional impairment; AP—physical activity; SRH—self-rated health; ^a^Exchange rate: $1 ∼ ¢4.8 as of the time of data collection.

Among those in the retirement cohort, more women were retired (53.2% [95CI = 45.8–60.5]) compared with men (46.8%, 95CI = 39.5–54.2) (*p* < 0.005). The retirees were more likely to be functionally impaired (44.7%, 95%CI 37.4–52.1) compared with those who were not retired (27.5%, 95%CI 24.7–30.3) (*p* < 0.001). Women were more likely to be functionally impaired (34.4%, 95%CI 31.0–37.9) than men (22.9%, 95%CI 19.1–27.1) (*p* < 0.001) (Table 2). Correlation analysis (Table 3) revealed that retirement (*r* = 0.136, *p* < 0.001), income (*r* = −0.143, *p* < 0.001), marital status (*r* = 0.126, *p* < 0.001) and psychological distress (*r* = 0.182, *p* < 0.001) were significantly associated with functional impairment following a Bonferonni correction for multiple correlations. Moreover, PA, age, gender, education, social connectedness, and SRH significantly related to both functional impairment and retirement.

**Table 2:** Bivariate distribution of demographic and health-related characteristics by gender and retirement status (*N* = 1201).

	Gender				Rao Scott χ^2^	Retirement status				Rao Scott χ^2^
	Men		Women			Not retired		Retired		
	%	(95%CI)	%	(95%CI)	*p*-value	%	(95%CI)	%	(95%CI)	*p*-value
Number	441		759			1012		188		
Total	36.8	(34.0–39.5)	63.2	(60.5–66.0)	–	84.3	(82.2–86.3)	15.7	(13.7–17.8)	–
**Age (in years)**										
50–59	34.9	(30.5–39.6)	35.8	(32.4–39.4)	0.478	40.7	(37.7–43.8)	7.4	(4.1–12.2)	< 0.001
60–69	30.6	(26.3–35.1)	27.4	(24.3–30.7)		27.1	(24.4–29.9)	36.7	(29.8–44.0)	
70 +	34.5	(30.0–39.1)	36.8	(33.3–40.3)		32.2	(29.3–35.2)	55.9	(48.4–63.1)	
**Retirement status**						–	–	–	–	–
Not retired	86.8	(84.2–89.2)	80.0	(76.0–83.7)	0.002	–	–	–	–	
Retired	20.0	(10.8–15.8)	13.2	(16.3–24.0)						
**Gender**										
Female	–	–	–	–	–	65.1	(62.1–68.1)	53.2	(45.8–60.5)	0.002
Male	–	–	–	–		34.9	(31.9–37.9)	46.8	(39.5–54.2)	
**PA**										
Inactive	38.8	(34.2–43.5)	55.7	(52.1–59.3)	< 0.001	47.5	(44.4–50.7)	60.1	(52.7–67.2)	0.002
Active	61.2	(56.5–65.8)	44.3	(40.7–47.9)		52.5	(49.3–55.6)	33.9	(32.8–47.3)	
**Residential status**										
Rural	47.8	(43.1–52.6)	43.3	(39.8–47.0)	0.131	45.4	(42.3–48.5)	43.1	(35.9–50.5)	0.565
Urban	52.2	(47.4–56.9)	56.7	(53.0–60.2)		54.6	(51.5–57.7)	56.9	(49.5–64.1)	
**Marital status**										
Married	71.1	(66.6–75.4)	29.1	(25.9–32.5)	< 0.001	44.1	(41.0–47.3)	45.7	(38.3–53.1)	0.075
Widow	12.7	(9.7–16.2)	55.9	(52.3–59.5)		41.4	(38.3–44.5)	34.2	(27.4–41.6)	
Divorced/separated	16.2	(12.8–20.0)	15.0	(12.5–17.7)		14.5	(12.4–16.9)	20.1	(14.6–26.6)	
**Level of education**										
Primary/Never	76.4	(72.2–80.3)	91.8	(89.7–93.7)	< 0.001	88.9	(86.8–90.8)	71.3	(64.2–77.6)	< 0.001
Secondary	13.6	(10.5–17.2)	5.8	(4.2–7.7)		7.9	(6.3–9.7)	12.8	(8.4–18.4)	
Higher	10.0	(7.3–13.2)	2.4	(1.4–3.7)		3.2	(2.2–4.4)	1.6	(1.1–2.2)	
**Income level (In Ghana Cedis)**										
Low	64.6	(59.7–69.3)	81.0	(77.7–83.9)	< 0.001	75.1	(72.1–77.9)	72.7	(64.8–79.6)	0.533
High	35.4	(30.7–40.3)	19.0	(16.1–22.3)		24.9	(22.1–27.9)	27.3	(20.4–35.2)	
**Social connectedness**										
Low	39.9	(35.3–44.6)	38.9	(35.4–42.4)	0.721	40.0	(37.0–43.1)	35.1	(29.3–42.4)	0.205
High	60.1	(55.4–64.7)	61.1	(57.6–64.6)		60.0	(56.9–63.0)	64.9	(57.6–71.7)	
**Self-rated health (SRH)**										
Excellent/Very Good	28.6	(24.4–33.0)	14.9	(12.4–17.6)	< 0.001	20.9	(18.5–23.6)	14.4	(9.7–20.2)	0.027
Good	30.6	(26.3–35.1)	30.8	(27.6–34.3)		31.2	(28.4–34.2)	28.2	(21.9–35.2)	
Fair	26.1	(22.0–30.4)	30.7	(27.4–34.1)		27.5	(24.7–30.3)	37.2	(30.3–44.6)	
Poor	14.7	(11.6–18.4)	23.6	(20.6–26.8)		20.4	(17.9–23.0)	20.2	(14.7–26.7)	
**Psychological Distress**										
Low	62.4	(57.7–66.9)	50.2	(46.6–53.8)	< 0.001	53.7	(50.5–56.8)	60.1	(52.7–67.2)	0.103
High	37.6	(33.1–42.3)	49.8	(46.2–53.4)		46.3	(43.2–49.5)	39.9	(32.8–47.3)	
**FI**										
Not impaired	77.1	(72.9–80.9)	65.6	(62.1–69.0)	< 0.001	72.5	(69.7–75.3)	55.3	(47.9–62.6)	< 0.001
Impaired	22.9	(19.1–27.1)	34.4	(31.0–37.9)		27.5	(24.7–30.3)	44.7	(37.4–52.1)	

FI—functional impairment; AP—physical activity; SRH—self-rated health.

**Table 3:** Correlations between the independent and dependent variables of interest.

Variable	FI	Retired
FI	1	.136***
Retired	.136***	1
PA	−.399***	−.091***
Age (in years)	.338***	.233***
Women	−.121***	.090***
Rural residence	.007	.017
Marital status: Not married	.126***	.020
Higher education	−.060**	.218***
Income level (in Ghana Cedis)	−.143***	−.017
Social connectedness	.101***	.115**
Suboptimal SRH	.390***	.057**
Psychological distress	.182***	−.047

Pearson product-moment correlations were used to calculate the association between continuous variables, point-biserial correlations were used to assess the relationship between continuous and dichotomous variables, and phi correlations were used to assess the relationship between dichotomous variables.

FI—functional impairment; AP—physical activity; SRH—self-rated health.

**Figure 2:**
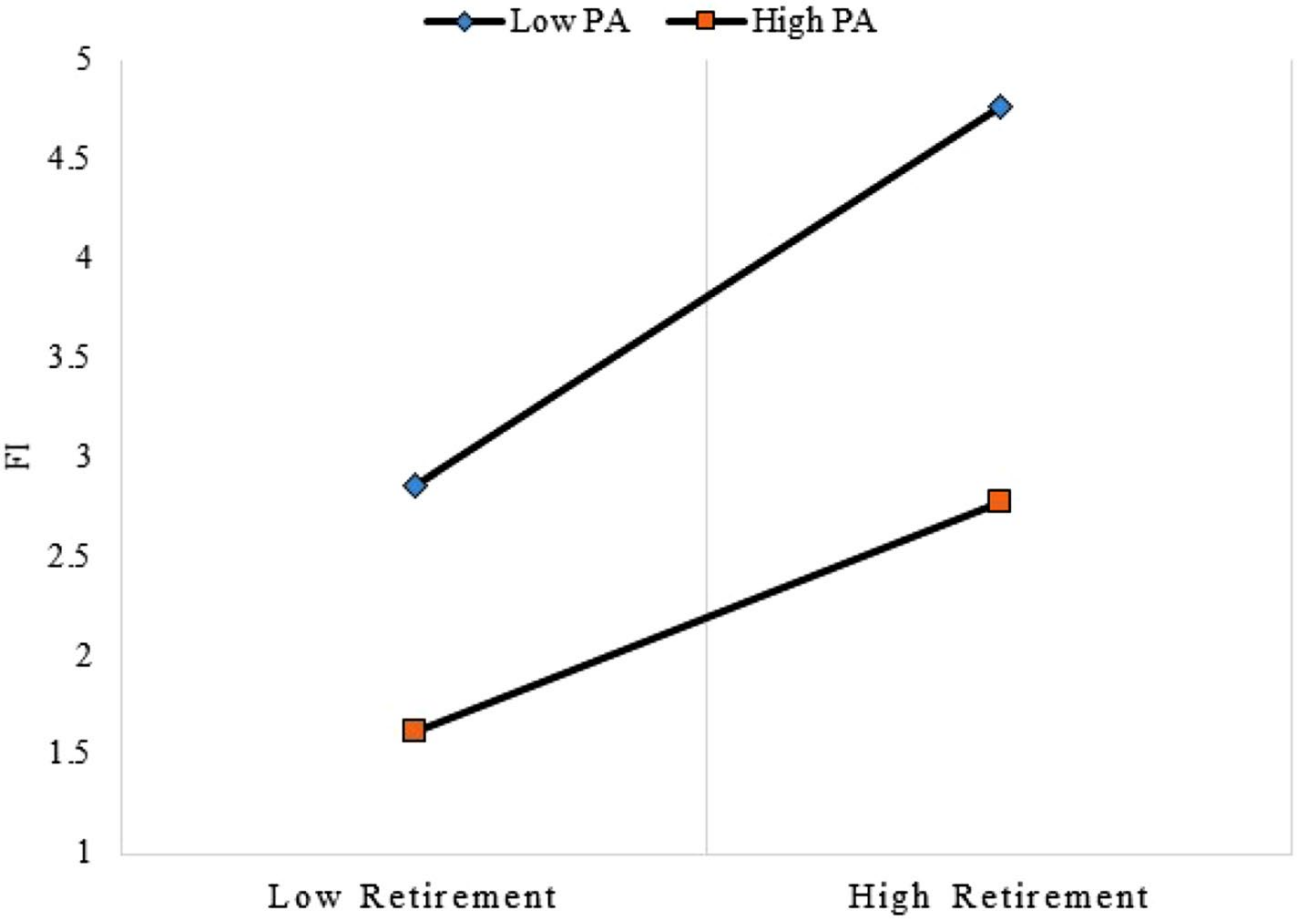
Physical activity (PA, the moderator) dampens the association of retirement with functional impairment (FI) in the overall sample.

**Figure 3:**
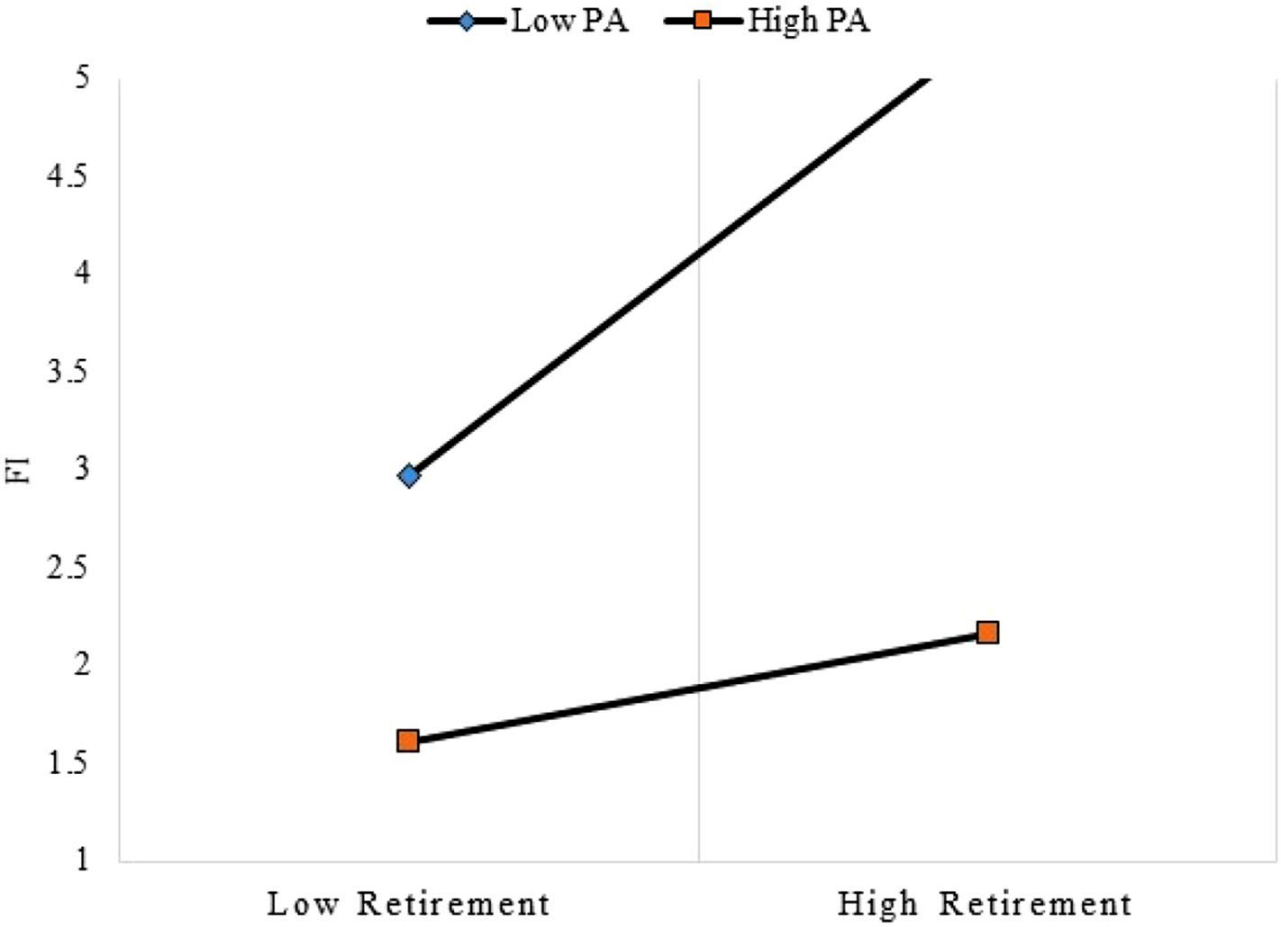
Physical activity (PA, the moderator) dampens the association of retirement with functional impairment (FI) in the ≥ 65 age group.

Table 4 shows the results of OLS regressions. Adjusting for potential confounders, retirement was significantly associated with increasing functional impairment in the total sample (*β* = 0.76, *p* < 0.001) and in men (*β* = 1.96, *p* < 0.001). In contrast, retirement was not associated with functional impairment in women (*p* = 0.075). Decreasing PA, worsening SRH, psychological distress, socially connected, increasing age, and being widowed were associated with an increased functional impairment. The second aim of this study tested the effect modification of the retirement-functional impairment association by PA. The main model was extended by adding the interaction term, retirement × PA. We found that PA moderated and dampened the relationship of retirement with functional impairment: retirement × PA predicted a decrease in functional impairment (*β* = −0.81, *p* < 0.005) (Fig. 2). In sensitivity analysis, we extracted age group ≥ 65 and estimated the effect of retirement on functional impairment (Table 5). Results showed slightly reduced estimates in terms of effect size: retirement increased functional impairment in the total sample (*β* = 0.71, *p* < 0.005), and in men only (*β* = 1.86, *p* < 0.001), but the effect was significantly moderated by PA (*β* = −0.43, *p* < 0.005) (Fig. 3). In a supplementary analysis, employment status was stratified into employed, unemployed, and retired. Retirement was positively associated with functional impairment with larger effect sizes in the total sample and men.

**Table 4:** Predicting FI Score with Retirement and PA Score: OLS Regression Models.

Variables	(1) Pooled Sample		(2) Male		(3) Female		(4) Interaction: Retirement × PA	
	β	(SE)	β	(SE)	β	(SE)	β	(SE)
Retired (Not retired)^a^	0.764	(0.254)***	1.955	(0.474)***	0.233	(0.328)	0.956	(0.251)***
PA score	−0.190	(0.023)***	−0.150	(0.045)***	−0.216	(0.028)***	−1.264	(0.229)***
Age (in years)	0.046	(0.009)***	0.076	(0.018)***	0.039	(0.010)***	0.045	(0.008)***
Gender (Male)^a^	1		1		1		1	
Female	0.281	(0.217)	–	–	–	–	0.182	(0.208)
Residential status (Rural)^a^	1		1		1		1	
Urban	0.065	(0.189)	−0.267	(0.384)	0.077	(0.227)	0.006	(0.184)
Marital status (Married)^a^			1		1		1	
Widow	0.453	(0.221)*	0.980	(0.448)*	0.271	(0.264)	0.502	(0.215)*
Divorced/separated	−0.492	(0.299)	−0.194	(0.535)*	−0.714	(0.388)	−0.619	(0.283)*
Education (Primary or none)^a^	1		1		1		1	
Secondary	−0.644	(0.387)	−1.426	(0.698)*	−0.459	(0.515)	−0.780	(0.386)*
Tertiary	−0.020	(0.415)	−0.565	(0.576)	−0.011	(0.711)	0.009	(0.396)
Income level (In Ghana Cedis)	−0.031	(0.242)	0.864	(0.470)	−0.181	(0.303)	−0.019	(0.237)
Social connectedness score	0.135	(0.067)*	−0.160	(0.132)	0.222	(0.082)**	0.250	(0.188)
SRH (Excellent/Very Good)^a^	1		1		1		1	
Good	0.036	(0.315)	−0.075	(0.573)	0.093	(0.389)	0.126	(0.309)
Fair	0.808	(0.298)**	1.342	(0.558)*	0.663	(0.370)	0.979	(0.295)**
Poor	2.004	(0.316)***	2.146	(0.567)***	1.934	(0.392)***	2.208	(0.314)***
Psychological Distress Score (10–50)	0.463	(0.187)*	1.094	(0.407)**	0.410	(0.224)	0.437	(0.180)*
*Interaction term*: Retirement × PA							−0.805	(0.457)**
Adjusted Pseudo R^2^	0.477		0.515		0.468		0.479	

β—Coefficients; SE—robust standard errors are presented in parentheses; SRH–Self-rated Health; ^a^Reference Group.

***p < 0.001; **p < 0.005; *p < 0.05.

**Table 5:** Predicting FI Score with Retirement and PA Score among ≥ 65 age group: OLS Regression Models.

Variables	(1) Pooled Sample		(2) Male		(3) Female		(4) Interaction: Retirement × PA	
	β	(SE)	β	(SE)	β	(SE)	β	(SE)
Retired (Not retired)^a^	0.707	(0.268)**	1.864	(0.478)***	0.019	(0.358)	0.773	(0.273)**
PA score	−1.114	(0.208)***	−1.024	(0.390)**	−1.268	(0.266)***	−1.004	(0.240)***
Age (in years)	0.044	(0.011)***	0.049	(0.022)*	0.042	(0.014)**	0.044	(0.011)***
Gender (Male)^a^	1		1		1		1	
Female	−0.022	(0.241)	–	–	–	–	−0.029	(0.242)
Residential status (Rural)^a^	1		1		1		1	
Urban	−0.058	(0.204)	−0.380	(0.407)	−0.123	(0.259)	−0.050	(0.205)
Marital status (Married)^a^			1		1		1	
Widow	0.769	(0.248)**	1.558	(0.517)**	0.451	(0.307)	0.451	(0.307)**
Divorced/separated	−0.610	(0.330)	0.047	(0.560)	−1.094	(0.462)*	−1.094	(0.462)
Education (Primary or none)^a^	1		1		1		1	
Secondary	−0.683	(0.438)	−1.706	(0.768)*	−0.239	(0.637)	−0.718	(0.441)
Tertiary	0.353	(0.469)	−0.569	(0.611)	0.989	(0.887)	0.312	(0.463)
Income level (In Ghana Cedis)	−0.060	(0.278)	1.181	(0.527)*	−0.375	(0.373)	−0.041	(0.279)
Social connectedness score	0.257	(0.215)	−0.302	(0.415)	0.427	(0.273)	0.259	(0.215)
SRH (Excellent/Very Good)^a^	1		1		1		1	
Good	−0.012	(0.349)	−0.322	(0.609)	−0.001	(0.448)	−0.019	(0.349)
Fair	0.894	(0.333)**	1.640	(0.592)**	0.527	(0.429)	0.908	(0.333)**
Poor	2.122	(0.357)***	2.240	(0.601)***	1.995	(0.458)***	2.149	(0.359)***
Psychological Distress Score (10–50)	0.513	(0.202)*	1.293	(0.432)**	0.277	(0.248)	0.518	(0.203)*
*Interaction term*: Retirement × PA							−0.431	(0.485)**
Adjusted Pseudo R^2^	0.400		0.510		0.371		0.401	

β—Coefficients; SE—robust standard errors are presented in parentheses; FI—functional impairment; AP—physical activity; SRH—self-rated health; ^a^Reference Group. ***p < 0.001; **p < 0.005; *p < 0.05.

## Discussion

### Main findings

This study from Ghana, an SSA country context, found a retirement rate of 15.7%, and 53.2% of the retirees were women. The prevalence of functional impairment was more common in women and retirees than in their counterparts. Independent of potential confounders, retirement was associated with increased functional impairment in a dose–response manner in the overall sample and in the men sub-group but not in women. The stratified analysis for the ≥ 65-year group observed a relatively reduced estimate. Additionally, PA moderated the relationship between retirement and functional impairment: the risk of impairment after retirement grew weaker for those engaged in PA. This moderation suggests that PA potentially attenuates the links between retirement and functional impairment. To our knowledge, this is the first study to estimate the effect modification of the association between retirement and functional impairment by PA and gender, particularly in the LMICs context. Although retirement remains inevitable, our findings emphasize the potential of PA in ensuring functional ability during retirement.

### Previous research and interpretation of the findings

As noted, research testing the association of retirement with functional impairment is limited in the SSA context, but our findings are aligned with prior studies reporting a positive retirement-functional impairment relationship in Western and Asian countries. For example, among 21,608 Australians from the 45 and Up Study, Byles et al.^40^ found a strong association between retirement and physical dysfunction over time. Analyzing data from the Health and Retirement Study (HRS) (*n* = 17,844), Stenholm et al.^19^ intimated that physical function declines faster in retirement than in full-time work. Dave et al.^14^utilized a sample of 77,194 adults and found that a 6-year average post-retirement caused a 5–16% increase in mobility and ADL difficulties and a 5–6% increase in illness conditions. Calvo et al.^41^ reported that early retirement dampens subjective physical health among Americans. Xue et al.^42^found that verbal memory function declined after retirement among 3,433 individuals from the Whitehall II Cohort Study. According to Hale et al.^43^, postponed retirement benefits cognitive function across diverse populations. However, our findings are inconsistent with previous studies suggesting better health outcomes during retirement^44,45^. In addition, Mein et al.^18^ in the Whitehall II longitudinal study, found no association of retirement at age 60 with physical health functioning.

Indeed, the physical health effects of retirement remains far from certain^13^. While some studies included small sample sizes^46^, others involved only specific adult population groups^47^. Crucially, the evidence from previous studies may not fully apply to the SSA context given the diversity of sociocultural, and demographic conditions, including aging dynamics. The current study builds on the previously published literature by providing new evidence of the effect modification of the association of retirement with functional impairment by PA and gender in the SSA population. Our findings may present important implications for relevant social policy and public health interventions aimed at improving functional independence and successful aging.

Retirement may be an important contributor to functional impairment through several plausible pathways. First, retirement may lead to disengagement and dissociation from an economic activity which, in turn, causes a feeling of alienation, powerlessness, social anxiety, and somatic disorders^48,49^. These mental stressors have been identified as key risk factors of functional limitations^21^. Second, having a satisfying job can be central to self-concept and personal identity^50^ and the exit from such jobs can make one feel lost through negative alterations in mood. This can potentially lead to a decline in perceived physical health^51^. Third, many people in SSA do not plan adequately for their retirement, largely due to unemployment, low-income levels, and the predominant contribution of the informal economic sector^37^. The transition to retirement could, therefore, spell shocks and plunge the retirees into potentially ongoing hopelessness and a feeling of uselessness. Retirement-induced sadness and emotional hyperarousal symptoms could neurologically damage physical functioning capacities, given that prior research has strongly linked mental distress in old age to functional impairment^52^.

Fourth, the paltry sum paid in many middle and lower-income and some high-income countries as pensions due to inadequate government/state pension schemes may contribute to the worsening economic well-being of retirees. Poor economic well-being has also been related to declining health status, including physical health^53,54^. Moreover, the retirement-induced role loss can dampen the hitherto, social networks and social interactions. Retirement, to a larger extent, shuts mobility and social connectedness which may lead to solitude, loneliness, and the likelihood of drug use. These circumstances, in turn, may contribute to poor physical health outcomes in old age^55^. Interestingly, as observed in our own study population, the magnitude of the effect of retirement on functional impairment appears to attenuate with advancing age. This suggests that older people might have developed coping strategies and become more resilient against the momentous retirement chocks over time.

Crucially, our analysis found effect modification of the association of retirement with functional impairment by PA. Thus, the engagement in PA after retirement was found to retard the risk of functional impairment. This observation is distinctive given that previous studies have not examined the interactive effect of PA and retirement on functional impairment. Prior research has shown that moderate-to-vigorous PA, which is likely less common after retirement, is associated with a lower risk of functional impairment^35^. As a health promotion mechanism, PA may potentially stimulate body and mind coordination to control physical functioning via neurotransmitters with a likely pathway that PA is a potent stimulus of immune function^56^. PA may also engender resourceful social interaction crucial to improving physical health outcomes, particularly in old age^30^.

Our study indicates that the majority of those who retired were women. This finding is similar to a recent report from the Longitudinal Social Protection Survey in which women were found to leave the labor market earlier than men among Latin American populations^25^. In addition, the risk of functional impairment after retirement differed by gender in specific subgroups. The association between retirement and functional impairment among women did was not robust. These observations are similar to results from a study in the USA that found differences in the retirement-cognitive decline association between men and women^43^. These findings might be, at least in part, due to women’s commonly low-status jobs and weaker attachment to the job market, particularly in SSA. Women are more likely to have  employment fragmented by longer career breaks, unemployment spells, part-time jobs, and have as a result fewer pensions than men^12,28,57^. In the African socio-cultural and traditional system, men are considered the breadwinners while women mainly perform family roles, including caregiving responsibilities. Women in many cases do not stay long in the job market, hence, retirement can have a little or no impactt on their physical health outcomes.

Our study utilized a large sample size and high-quality data from a representative survey. This allowed the unique opportunity to analyze the effect modification of the association of retirement with functional impairment by PA and gender in Ghana for the first time. Despite these strengths, the study has several limitations. Although we hypothesized retirement to influence functional impairment, the present study cannot determine causality, and it is important to note that the associations of retirement and functional impairment are most likely bidirectional. Future studies might, usefully consider longitudinal analysis to replicate or validate these relationships. Although rigorous adjustments in regression models were undertaken, we were unable to rule out potential confounding incidence by unobserved factors. It should also be noted that the measurement of retirement, functional impairment, and PA was based on self-report which had not been validated in the study context. Recall bias is also inevitable.

### Public health and policy implications

The findings from the present study contribute to the published literature on the physical health impacts of retirement. Our study also provides important implications for policy and public health interventions targeted at improving physical health outcomes during retirement, and old age in general. Policymakers should make concerted efforts to enact policies to prolong the retirement age and also offer near-retirement work incentives to encourage working beyond a certain limit, particularly among men who may have the capacity to work. In addition, measures to promote physical health in later life should be implemented during retirement and often planned and initiated well in advance of retirement. Creating the enabling environment for regular effective PA and exercise modules for older people may be desirable in retarding or delaying the onset of functional impairments after retirement^30^. Policies that encourage PA would also be potent in buffering the retirement-induced sedentary lifestyle, immobility, loneliness, and their concomitant functional health effects. Promoting functional health and well-being in retirees may be of utmost importance, and this approach may consist of public education programs, clinical therapy, and behavioral change interventions.

## Conclusions

This representative study from the SSA context has shown that functional impairment in old age, particularly among men increases after retirement. Although retirement might be one promising risk factor for physical health overall, the association non-existed in women. The findings provide additional evidence that the adverse health effects of retirement are mitigated if older persons continue to engage in PA post-retirement. This indicates that work may be underscored as a fundamental form of PA for many older people. Future research would benefit from replicating the current study using more longitudinally robust data in other settings and the potential mediating variables in the association.

## Methods

### Participants

Data for this analysis came from the AgeHeaPsyWel-HeaSeeB, a nationally representative sample of community-dwelling adults aged ≥ 50 years. The original study investigated the specific impacts of the sociopolitical, informal social support, and socio-demographic inequalities on the health, well-being, and health-seeking options of older people in Ghana^58^. The design was based upon a multi-stage stratified cluster sampling of households within representative statistical areas delineated by geographical and socio-demographic criteria. Briefly, the area was demarcated into three sub-regional sectors based on geographic and locational uniqueness, and two districts were randomly selected from each sub-regional zone. The selected districts were demarcated into rural and urban areas based on the GSS's^37^ classification; nine urban and 15 rural neighborhoods were randomly selected.

We estimated the sample size (*N*) following Lwanga et al.^59^: *N* $$= design\,effect\, \times \,\left[ {\left( {Z_{{{\raise0.7ex\hbox{$\alpha $} \!\mathord{\left/ {\vphantom {\alpha 2}}\right.\kern-\nulldelimiterspace} \!\lower0.7ex\hbox{$2$}}}} } \right)^{2} \times P\left( {1 - P} \right)} \right]/\varepsilon ^{2}$$. We assumed a 5% margin of error, 95% confidence interval, design effect of 1.5, 5%, and 15% of type 1 and type 2 errors respectively, and a conservative prevalence of 50% given that the actual proportion of ≥ 50 olds was unknown. This sample achieved a statistical power of 85% and 5% (two-sided) significance level to detect an odds ratio of ≥ 2 and the required minimum sample size was 901. We oversampled by 28% to cater for potential non-responses and to improve generalizability. Therefore, 1247 older persons were recruited via systematic random sampling technique. We excluded those who were not available during data collection (*n* = 17) and those who declined to participate (*n* = 11). Final analyses were performed for 1201 older adults after excluding participants whose essential data were missing (*n* = 15) or contained outliers (*n* = 3) (Fig. 1). Data were collected by trained interviewers and informed written or oral consent was obtained from all participants. The study protocol received ethics approval from the Committee on Human Research Publication and Ethics, School of Medical Sciences, Kwame Nkrumah University of Science and Technology, and Komfo Anokye Teaching Hospital, Kumasi, Ghana (Ref: CHRPE/AP/507/16). In addition, the research ethics committee of Lignan University approved the study and all the procedures involved in this study. The methods were performed taking into account the relevant guidelines, standards, and regulations.

### Measures

#### Gender and potential confounders

Gender differences were self-reported as male or female. Other demographic covariates included age (in years), residence (rural, urban), and marital status (currently married, widowed, divorced/separated). Socioeconomic covariates included education reflected the highest obtained education (primary or none, secondary, tertiary), income (in Ghana Cedis), social connectedness/participation reflected past 30-day involvement, and social participation in the community (never, less frequently, frequently, very frequently, every day). The overall score ranged from 0 to 20 with a higher score reflecting higher social connectedness. Self-rated health was assessed with a single-item measure of the Short Form Health Survey 36 Questionnaire (very good, good, fair, and poor)^60^. Mental health was measured by the Kessler Psychological Distress Scale (K-10) (none of the time, a little of the time, some of the time, most of the time, all of the time)^20^ with a sum score ranging from 0 to 40 with higher scores reflecting higher psychological distress outcomes.

#### Functional impairment

Self-reported difficulty in conducting ADL in the preceding 30 days commonly used to gauge older people’s daily performance^61^ with good psychometric properties^62^ was used as proxies to assess functional impairment. These were based on the six items on eating, bathing or washing the whole body, dressing up or putting on clothes, getting in or out of bed, reaching and using the toilet, and moving around inside your house. These responses were measured on a four-point scale ranging from (0) not limited at all (1) less limited (2) somewhat limited and (3) much limited. The total score ranged from 0 to 18 with a higher score indicating greater functional impairment. The physical function scale has strong validity and internal consistency (*α* = 0.83).

#### Retirement

Retirement is defined as being out of the labor force with no intent to seek employment in the future. Based on this definition, the study sample was considered as retired when they had not been working for pay for at least one year^63^, and they self-reported that they retired at the time of the interview^9^. This reflected a self-reported retirement status or self-reported employment status. The analysis focuses on retirees rather than non-workers as many published literature refers exclusively to the former. Prior research used a similar approach with the rationale that being out of the labor force does not instantaneously affect health status^63^. The retirement status variable was, therefore, constructed to indicate whether individuals were retirees (those who had not worked for at least a year and considered themselves as retired) (coded 1) or non-retirees (those working for pay, had not worked for less than a year, and/or did not consider themselves as retired) (coded 0).

#### Physical activity (PA)

PA was assessed using the International Physical Activity Questionnaire short form (IPAQ-SF). The IPAQ-SF is a validated screening tool that measures three dimensions of sitting time and PA intensity (low, moderate, and vigorous) over the past seven days as part of the daily activities of adults. The IPAQ-SF is used to calculate total physical activity in metabolic equivalent (MET) energy expenditure-min per week^64^ Respondents were asked: “During the last 7 days, on how many days (1) …did you walk for at least 10 min at a time including walking at work, at home, and to travel from place to place? (2) …did you do moderate physical activities like gardening, cleaning, bicycling at a regular pace, swimming, or other fitness activities? (3) …did you do vigorous physical activities like heavy lifting, digging, heavier garden, or construction work, chopping woods, aerobics, jogging/running, or fast bicycling?” The responses were taken on a continuous scale. The higher the score the higher the level of PA. The IPAQ-SF has been validated in the older African population with good reliability/validity^65^. The physical activity scale has strong validity and reliability (*α* = 0.89).

### Statistical analysis

Descriptive analyses were first performed to describe the sample and reported as means and standard deviations for continuous variables, or counts and percentages for categorical variables. We performed Pearson’s zeroth-order correlations of relevant exposure variables with functional impairment outcome. The *p*-value was adjusted for the multiple correlations, which can increase the risk of a type I error, i.e., to erroneously conclude the presence of a significant correlation. Rao Scott contingency table *χ*^2^ statistics were used for simple comparisons of categorical variables and a student *t*-test was performed for continuous variables between gender subgroups and retirement status. The 95% confidence intervals (CI) for each variable category were estimated.

A three-stage OLS model was built in which the ADL score was regressed on the study variables to test the adjusted effect of retirement on functional impairment outcome. First, the functional impairment score was regressed on the retirement status in the full sample adjusting for potential covariates. Next, stratified analysis by gender was conducted to investigate whether the effect of retirement on functional impairment could be tempered by gender differences. Finally, we included the interaction term, retirement × PA, in the overall sample to evaluate the effect modification of the association of retirement with functional impairment by PA. In addition, a simple slope test was performed by testing the conditional effects at one SD above and below the mean when the interaction effect reached significance. The auxiliary analysis considered a within-employment status variability modeling. Thus, we stratified the sample into three employment cohorts: employed (44.4%), unemployed (39.9%), and retired (15.7%) to understand any employment-related differences in the effect of retirement on functional impairment. Cluster-robust standard errors were calculated. We checked for multicollinearity by computing the Variance Inflation Factor (VIF) but the VIF scores ranged ≤ 1.3, indicating no multicollinearity. All analyses were conducted using IBM-SPSS V.25 Software and the level of significance was *p* < 0.05 (two-tailed).

### Ethical approval

The study protocol received ethics approval from the Committee on Human Research Publication and Ethics, School of Medical Sciences, Kwame Nkrumah University of Science and Technology, and Komfo Anokye Teaching Hospital, Kumasi, Ghana (Ref: CHRPE/AP/507/16).

### Informed consent

Written informed consent was obtained from all individual participants included in the study.

## Data Availability

Those interested in the data and materials in this paper should contact the corresponding authors, Razak M. Gyasi at the email address RGyasi@aphrc.org.

## Abbreviations

ADL: Activities of daily living
CHRPE: Committee on Human Research Publication and Ethics
CI: Confidence intervals
GSS: Ghana Statistical Service
HRS: Health and Retirement Study
IPAQ-SF: International Physical Activity Questionnaire short form
LMICs: Low- and middle-income countries
MET: Metabolic equivalent
OLS: Ordinary least squares
PA: Physical activity
SRH: Self-rated health
SSA: Sub-Saharan Africa
UN: United Nations
VIF: Variance Inflation Factor

## Code availability

Data reported in this study is not freely available online but would be made available upon request.
